# Utilization of dried blood spot specimens can expedite nationwide surveillance of HIV drug resistance in resource-limited settings

**DOI:** 10.1371/journal.pone.0203296

**Published:** 2018-09-07

**Authors:** Guoqing Zhang, Joshua DeVos, Sandra Medina-Moreno, Nicholas Wagar, Karidia Diallo, R. Suzanne Beard, Du-Ping Zheng, Christine Mwachari, Carolyn Riwa, Boniface Jullu, Ngugi Evelyn Wangari, Mary S. Kibona, Lucy W. Ng'Ang'A, Elliot Raizes, Chunfu Yang

**Affiliations:** 1 International Laboratory Branch, Division of Global HIV & TB, Center for Global Health, CDC, Atlanta, GA, United States of America; 2 Institute of Human Virology, University of Maryland School of Medicine, Baltimore, MD, United States of America; 3 CDC Kenya, Nairobi, Kenya; 4 The Ministry of Health Tanzania, Dar es Salaam, Tanzania; 5 CDC Tanzania, Dar es Salaam, Tanzania; 6 Adult Care and Treatment Branch, Division of Global HIV & TB, Center for Global Health, CDC, Atlanta, GA, United States of America; National and Kapodistrian University of Athens, GREECE

## Abstract

**Introduction:**

Surveillance of HIV drug resistance (HIVDR) is crucial to ensuring the continued success of antiretroviral therapy (ART) programs. With the concern of reduced genotyping sensitivity of HIV on dried blood spots (DBS), DBS for HIVDR surveillance have been limited to ART-naïve populations. To investigate if DBS under certain conditions may also be a feasible sample type for HIVDR testing in ART patients, we piloted nationwide surveys for HIVDR among ART patients using DBS in two African countries with rapid scale-up of ART.

**Methods:**

EDTA-venous blood was collected to prepare DBS from adult and pediatric ART patients receiving treatment during the previous 12–36 months. DBS were stored at ambient temperature for two weeks and then at -80°C until shipment at ambient temperature to the WHO-designated Specialized HIVDR Laboratory at CDC in Atlanta. Viral load (VL) was determined using NucliSENS EasyQ® HIV-1 v2.0 kits; HIVDR genotyping was performed using the ATCC HIV-1 Drug Resistance Genotyping kits.

**Results:**

DBS were collected from 1,368 and 1,202 ART patients; 244 and 255 these specimens had VL ≥1,000 copies/mL in Kenya and Tanzania, respectively. The overall genotyping rate of those DBS with VL ≥1,000 copies/mL was 93.0% (95% CI: 89.1%-95.6%) in Kenya and 91.8% (87.7%-94.6%) in Tanzania. The turnaround times for the HIVDR surveys from the time of collecting DBS to completing laboratory testing were 6.5 months and 9.3 months for the Kenya and Tanzania surveys, respectively.

**Conclusions:**

The study demonstrates a favorable outcome of using DBS for nationwide surveillance of HIVDR in ART patients. Our results confirm that DBS collected and stored at ambient temperature for two weeks, and shipped with routine courier services are a reliable sample type for large-scale surveillance of acquired HIVDR.

## Introduction

The accessibility of HIV antiretroviral therapy (ART) for HIV-infected persons has substantially increased worldwide in recent years. According to the World Health Organization (WHO) statistics, about 20.9 million people were receiving ART globally as of June 2017. About 19.2 million are in low- and middle-income countries representing a 46-fold increase from since 2003 [1]. The expansion of ART programs has led to drastic reductions in HIV-related morbidity and mortality. However, as the access to ART continues to expand rapidly worldwide, often without adequate virological monitoring of patients on ART, emergence and transmission of HIV drug resistance (HIVDR) is a valid concern [2]. Studies in resource-limited settings have suggested that transmitted HIVDR in recently HIV-infected populations or pre-treatment HIVDR (PDR) in ART eligible patients is rising in countries where access to ART has been expanded [3–11], while acquired HIVDR (ADR) in patients on ART has been detected in majority of the patients failing ART [4, 9, 12–16].

It is well known that HIV can develop ADR mutations when inadequate blood drug concentrations occur due to treatment interruptions, poor adherence, or the use of suboptimal drug regimens [17]. While routine testing for HIVDR remains unavailable to the majority of patients receiving ART in resource-limited settings, population-based surveillance of ADR is essential to ensure the efficacy of ART regimens, assess the emergence of HIVDR, and understand the patterns of HIVDR in patients failing 1^st^-line or 2^nd^-line regimens so to switch to efficacious 2^nd^ line or 3^rd^ line regimens [18].

From 2004 to 2012, prospective surveys of ADR were implemented in at least 13 countries using the WHO-recommended ADR survey methodology [19]. However, implementation of prospective ADR surveys had met challenges because the prospective survey methodology requires a period of 12–15 months of follow-up [20]. While the WHO prospective survey can provide comprehensive results, the methodology requires extended time along with the high cost for plasma specimen collection and shipment, which limits its implementation and has made assessment of ADR at large scale difficult.

To address these limitations, WHO in collaboration with the Centers for Disease Control and Prevention (CDC) and the United States President's Emergency Plan for AIDS Relief (PEPFAR) developed a simpler cross-sectional survey to assess ADR [20]. The new survey method is designed to have a shorter specimen collection period and is easier to implement in a large number of ART clinics using national representative sampling, thus it can provide a timely description of ADR within countries scaling up of ART [20].

Although dried blood spots (DBS) have been routinely used for early infant diagnosis of HIV in resource-limited countries [21], due to the concern of reduced genotyping sensitivity with DBS their usage in HIVDR genotyping has been limited to surveillance of transmitted DR in ART-naïve populations or surveillance of ADR at baseline time point when patients were initiating ART [22]. However, findings from our studies and other investigators in resource-limited settings on DBS for VL measurement and DR testing have provided evidence that DBS may be a viable alternative sample type for VL and ADR monitoring in ART patients when appropriate VL testing platforms and more sensitive genotyping assays, such as ATCC kits, are used for HIVDR testing [23–33]. In addition, a recent study has further investigated the optimal conditions for DBS storage and shipment at ambient-temperature for HIVDR genotyping [26].

To inform updated methodologies for cross-sectional ADR surveillance, and to test the feasibility of using DBS for large-scale HIV VL testing and DR genotyping, we conducted two pilot ADR surveys in Kenya and Tanzania using nationally representative samples in a cross-sectional survey design. According to the 2013 UNAIDS Report on the Global AIDS Epidemic at the time when the surveys were conducted, Kenya and Tanzania had estimated 3.1 million people living with HIV in 2012 [34]. Despite widespread scale-up of ART services in Kenya and Tanzania [35], implementation of national surveillance of ADR had been limited in these two countries. This is due in part to the challenges with sampling method requirement of ensuring proper collection, shipment and storage of plasma samples. Nationwide surveillance of ADR is imperative to provide timely critical information to assess the performance of first-line regimens in the country, and inform the national ART program to select optimal second-line regimens, thereby maximizing the efficiency of ART programs.

Here we report the performance of DBS sample type-based strategies in the two pilot nationwide surveys of HIVDR. Our results confirm that DBS collected under certain condition are a feasible sample type for ADR survey. Results on virological outcome and DR patterns among patients in the surveys had been presented elsewhere [36–38].

## Materials and methods

### Study site selection

Ethical Review Committees of the Kenya Medical Research Institute (KEMRI), the Tanzania National Institute for Medical Research (NIMR) and Institute of Human Virology of University of Maryland School of Medicine approved the protocols. The anonymous testing at CDC was determined as non-human subjects research by the Office of the Associate Director for Science at the Center for Global Health, CDC, Atlanta, GA, USA.

Following the WHO protocol for cross-sectional surveillance of acquired HIV drug resistance [20], fifteen sentinel sites were selected using stratified sampling method for participation in each country. Sites were selected reflecting the bulk of where ART services were delivered and the heterogeneity of ART sites in each country. The goal of the sampling procedure was to select sites that were representative of the National ART Program in Kenya and Tanzania. The site selection reflected nationally important fixed effects, such as geographic region and size, as well as programmatic effects, such as facility level (i.e. national reference hospital/provincial general hospital/district hospital/sub-district hospital/health center/other hospital types) and administration type (i.e. public/non-public).

### Enrollment of participants

Two adult cohorts and one pediatric cohort were included in the survey from each country according to the WHO protocol. The inclusion criteria for each of the cohorts were as follows: (1) HIV-infected adults (age 15 and up) with documented ART start dates who had been on ART for at least 12 months and no more than 15 months; (2) HIV-infected adults (age 15 and up) with documented ART start dates who had been on ART for at least 24 months and no more than 36 months; (3) HIV-infected children (age 1 to 14 years) with documented ART start dates who had been on ART for at least 12 months and no more than 36 months. Patients presenting to the selected clinics for their regular clinic appointments and meeting inclusion criteria were asked to participate in the study. After obtaining informed written consent, they were consecutively enrolled into the study until the planned sample size for each site was reached. At enrollment, demographic, clinical and ART data were also abstracted from the medical records from each of the consented patients.

### DBS collection, handling, processing, and tracking

**Fig 1:**
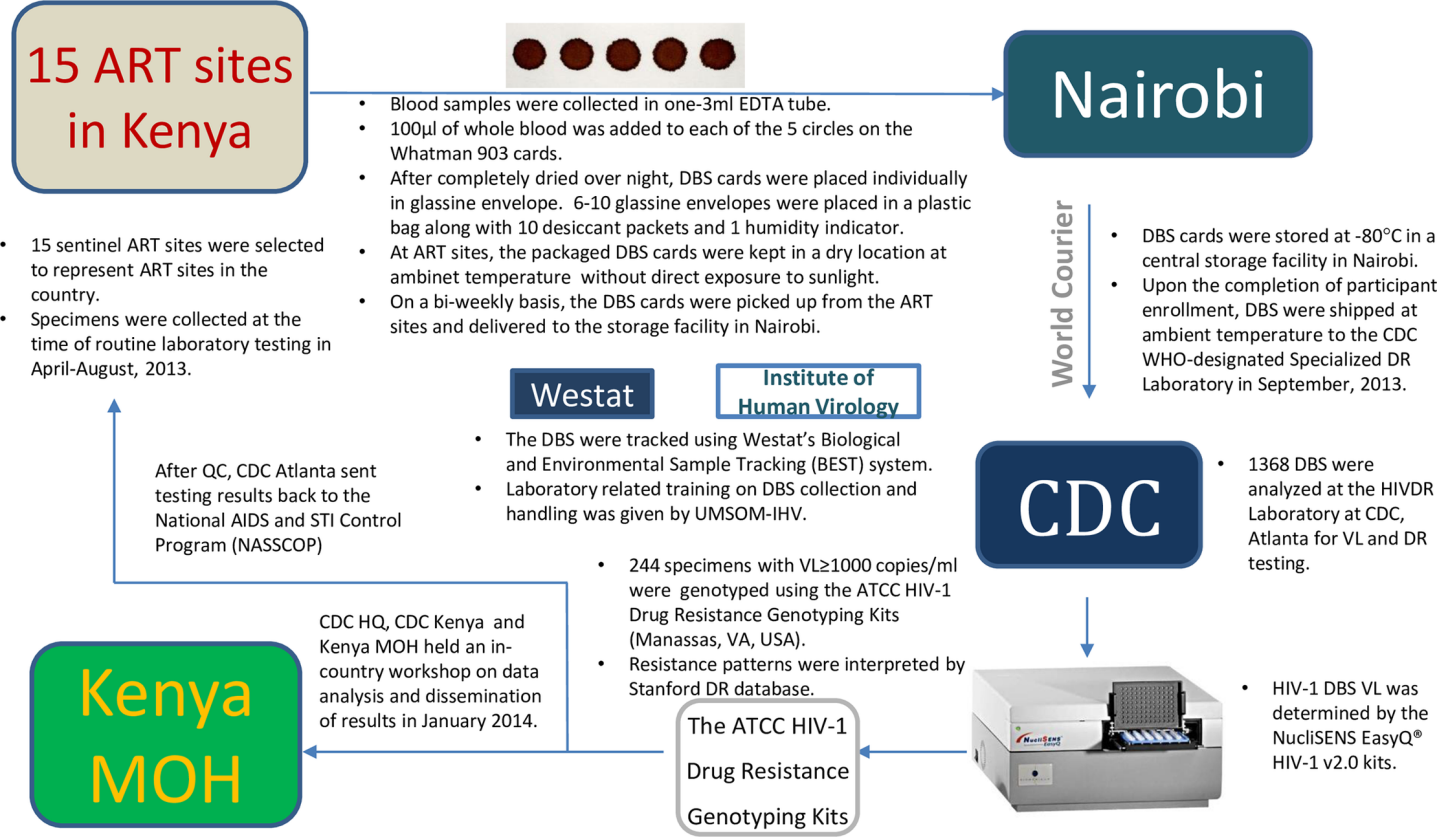
DBS specimens and data flow diagram in nationwide surveillance of acquired HIVDR, Kenya, 2013.

Blood from each patient was collected in a 3-mL vacuum tube containing ethylene diamine tetra-acetic acid (EDTA) anticoagulant and used for preparation of two DBS cards at the ART clinics. DBS were made following a standardized protocol for ATCC kits by dropping of 100 μl of whole blood to each of the five pre-printed circles in the Whatman 903 card, dried overnight and then packaged with desiccants and a humidity indicator card. At the clinics, the dried and packaged DBS cards were kept in a dry and safe location at ambient temperature with no direct exposure to sunlight. The DBS cards were picked up biweekly from the ART sites by routine courier services and delivered to the storage facility in Nairobi or Dar es Salaam where they were stored at -80°C until shipment. Upon the completion of DBS collection, DBS were shipped at ambient temperature to the drug resistance genotyping laboratory at the International Laboratory Branch, U.S. CDC in Atlanta, Georgia for processing. Fig 1 is a schematic representation of the process for the Kenya surveys.

### Nucleic acid extraction and HIV-1 VL analysis

One DBS spot was cut out per specimen and placed in a tube containing 2 mL of NucliSENS lysis buffer (Biomerieux, Durham, NC) for 30 minutes at room temperature with gentle rotation. Nucleic acid was then extracted using the NucliSENS® easyMAG® (Biomerieux) automated extraction system according to the manufacturer’s instructions. Nucleic acid was eluted in 25 μL of NucliSENS Extraction Buffer 3 and used for downstream testing. HIV-1 VL was measured by the NucliSENS EasyQ® automated system using NucliSENS EasyQ® HIV-1 v2.0 RUO test kits (Biomerieux) following the manufacturer’s instructions. For the specimens giving invalid result on the first test, a repeat test was performed, if an invalid result was produced again, no further attempt was made. With the initial input of 100 μl of whole blood, the assay has a limit of detection of 802 copies/mL for DBS and a linear range of detection of 500–21,000,000 copies/mL [39].

### Drug resistance genotyping

Genotyping of the protease and reverse transcriptase regions of the HIV-1 *pol* gene was performed for the specimens from patients with virological failure (defined as VL ≥1,000 copies/mL) using the ATCC^®^ HIV-1 Drug Resistance Genotyping Kit (ATCC, Manassas, VA). The ATCC kit was developed based on a broadly sensitive in-house genotyping assay developed by CDC [40]. In brief, a 1084 base-pair segment of the 5’ region of the *pol* gene was generated by RT-PCR and nested PCR using the kit Module 1: RT-PCR & Nested PCR (ATCC® GK-0098™). The purified PCR fragment was then sequenced using the kit Module 2: Cycle Sequencing (ATCC® GK-0200™), and the sequencing reactions were analyzed on the ABI Prism™ 3730 Genetic Analyzer (Applied Biosystems). The customized ReCALL (version 2.25) software program was used to edit the raw sequences and generate consensus sequences [41]. REGA HIV-1 Subtyping Tool version 3 [42] was used to determine HIV-1 subtypes. For those sequences with unresolved results by REGA Subtyping Tool, a phylogenetic analysis was conducted using neighbor-joining method and distance analysis in BioEdit [43] with the HIV-1 reference subtypes and circulating recombinant forms (CRFs) downloaded from the Los Alamos database [44].

### Statistical analysis

The prevalence (or proportion) estimates and their respective 95% confidence intervals (CIs) were calculated and adjusted for sample design through complex sample analysis. The complex sample analysis plan was defined based on the clusters of sampling protocol, strata (clinic type and clinic size) and the determined weights. The rates and proportions between groups were compared using the chi-square test, and the comparison of the groups of ordered classified data was performed by the Ridit analysis. Statistical calculations were performed using SPSS (Statistical Package for the Social Sciences) software (version 21.0; IBM, Armonk, NY). All tests were 2-tailed and a P<0.05 was considered statistically significant.

The sequences generated in the study have been submitted to GenBank with the accession numbers of MH317297—MH317524 for the Kenya samples and MH317525—MH317758 for the Tanzania samples.

## Results

### Demographic and clinical characteristics of participants

A total of 1,368 (438 adults on ART for 12–15 months, 468 adults on ART for 24–36 months, and 462 children on ART for 12–36 months) HIV-1 infected subjects receiving ART at 15 clinical sites in Kenya were enrolled in this study. A total of 1,202 (361 adults on ART for 12–15 months, 442 adults on ART for 24–36 months, and 399 children on ART for 12–36 months) HIV-1 infected subjects receiving ART at 15 clinical sites in Tanzania were enrolled in this study. For pediatric populations, the median age of the participants from both countries was seven years.

### Specimen handling

The dried and packaged DBS cards were stored at ambient temperature for an average of 7 days (range from one to 14 days) and 11 days (range from two to 42 days) before they were transported and stored in the -80°C freezers at the central storage facility in Kenya and Tanzania, respectively. Overall, 10.9% (279/2,569) of the DBS cards were stored at ambient temperature for over two weeks, with median length of 19 days (IQR, 16–22 days). All the DBS specimens from Kenya were stored at ambient temperature within two weeks.

### Viral load measurement

Viral load measurements were performed for all 2,570 DBS specimens collected from Kenya (N = 1,368) and Tanzania (N = 1,202) using NucliSENS EasyQ® HIV-1 v2.0 RUO test kits. At the first test run, 93.7% (2,409) of the DBS yielded valid VL results while 6.3% (161) were invalid due to inhibition of amplification of the internal control. Upon repeated test, all but one specimen from Kenya had valid VL measurements. Of the 1,367 specimens from Kenya with VL results, 244 had VL ≥1,000 copies/mL while among the 1,202 specimens with VL data available in Tanzania, 255 specimens had VL ≥1,000 copies/mL.

### Drug resistance genotyping and impact of VL levels on drug resistance genotyping

**Fig 2:**
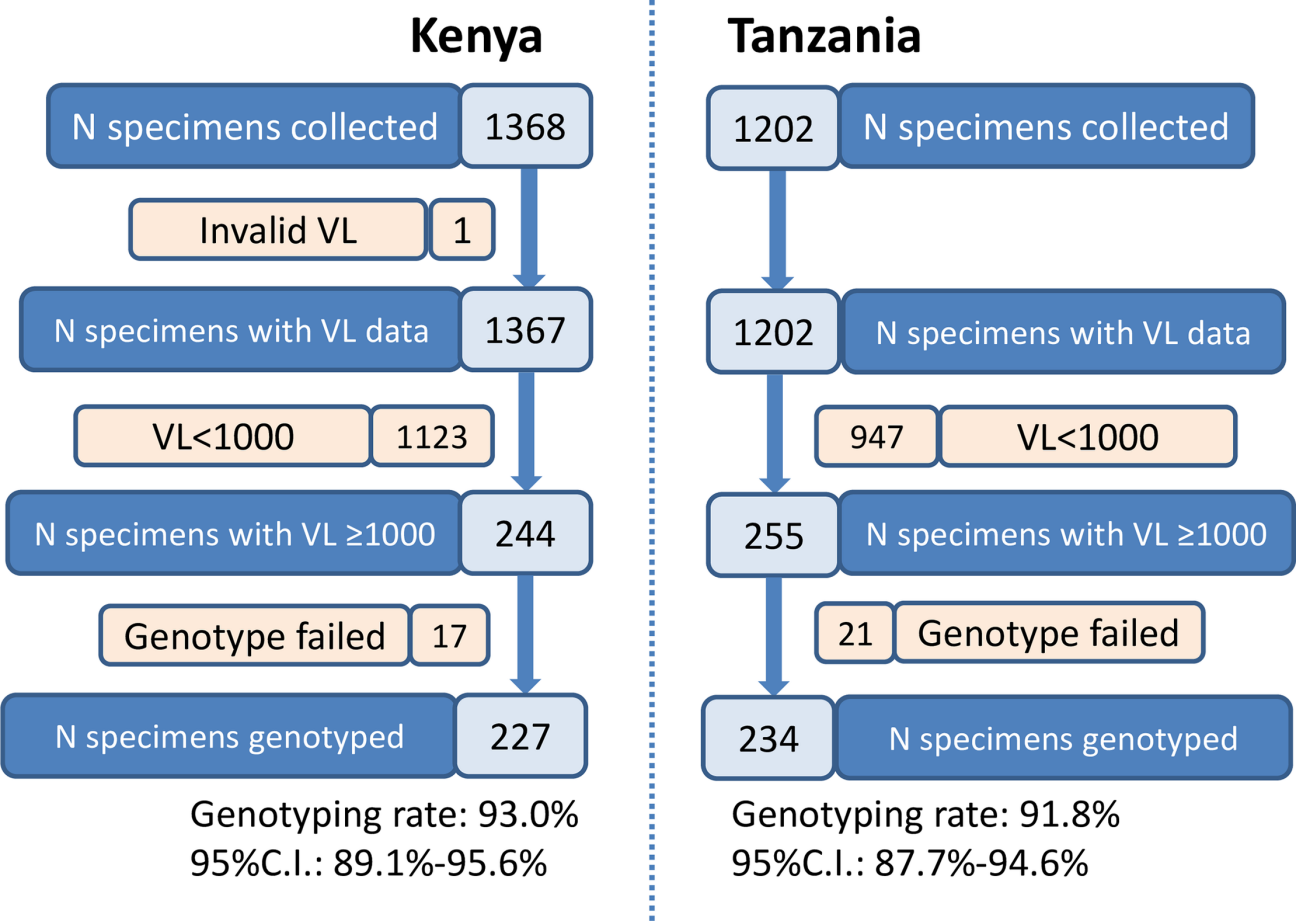
Flow diagram of VL measurement and drug resistance genotyping in nationwide surveillance of acquired HIVDR in Kenya and Tanzania, 2013.

With all the DBS with VL ≥1,000 copies/mL genotyping was performed using ATCC HIV genotyping kits. For DBS collected from Kenya, 93.0% (227/244, 95% CI: 89.1%-95.6%) of the specimens were genotyped while 91.8% (234/255, 95% CI: 87.7%-94.6%) of the DBS specimens collected from Tanzania were successfully genotyped (Fig 2).

**Fig 3:**
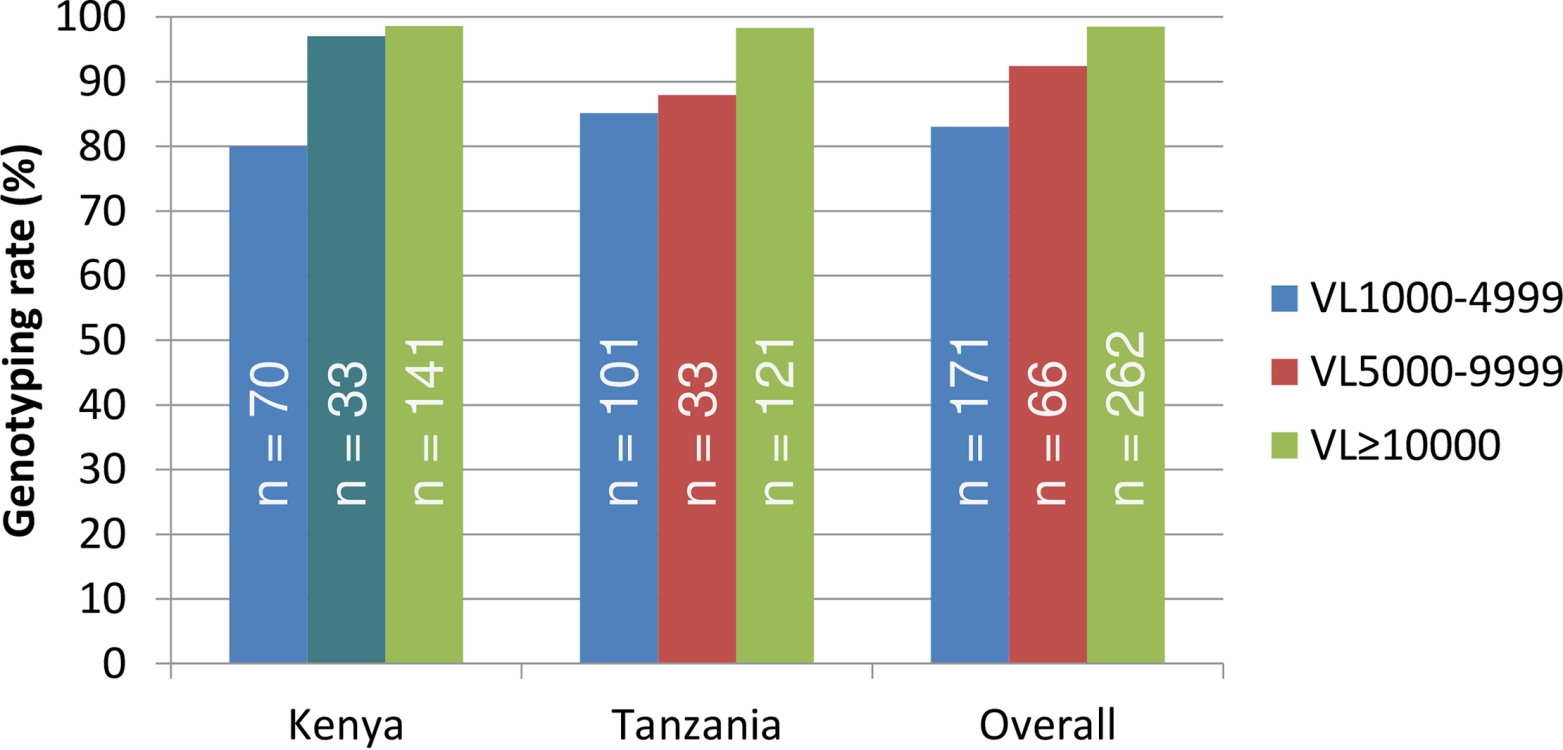
HIVDR genotyping rates at different VL level categories in nationwide surveillance of acquired HIVDR in Kenya and Tanzania, 2013.

To analyze the influence of VL levels on DR genotyping rates, we combined the VL data from both countries and stratified them into 1,000–4,999, 5,000–9,999 and ≥10,000 copies/mL categories. As expected, an overall positive relationship between VL levels and DR genotyping rates were observed (*P* < 0.001, Ridit analysis). In Kenya and Tanzania, 80.0% and 85.1% of the DBS specimens with VL 1,000–4,999 copies/mL were genotyped; 97.0% and 87.9% with VL 5,000–9,999 copies/mL were genotyped; 98.6% and 98.3% with VL ≥10,000 copies/mL were successfully genotyped, respectively (Fig 3). No significant difference was observed in genotyping rates between Kenya and Tanzania specimens (*P* = 0.59). Of the 298 specimens from pediatric patients with VL ≥1,000 copies/mL, 277 were genotyped (93.0%); and among the 201 specimens from adult patients with VL ≥1,000 copies/mL, 184 (91.5%) were genotyped (*P* = 0.56 for any significant difference).

To further analyze the impact of DBS quality on genotyping rates, we analyzed the storage length on genotyping rates. Among the 499 specimens collected from virologic failure patients, 75 specimens were stored at ambient temperature at the clinics for over two weeks, 67 (89.3%) of them were successfully genotyped while among the 424 specimens collected from virologic failure patients that were stored at ambient temperature at the clinics sites for less than or equal to two weeks, 394 (92.9%) of them were successfully genotyped. Although a small difference was noted there was no significant difference in genotyping rate between the two groups of specimens (χ^2^ = 1.17, P = 0.28).

### Distribution of HIV-1 subtypes among the newly obtained sequences

We analyzed the HIV-1 subtype and CRF distributions among the newly obtained 461 sequences using REGA Subtyping Tool and BioEdit [43] along with HIV-1 reference subtypes and CRFs downloaded from Los Alamos database. The distribution of subtypes, CRFs and unique recombinant forms (URFs) is as follows: subtype A1, 229 (49.7%); subtype C, 109 (23.6%); subtype D, 78 (16.9%); subtype A2, 1 (0.2%); subtype B, 1 (0.2%); CRF_A1D, 12 (2.6%); CRF_A2D, 8 (1.7%); CRF_CD, 3 (0.7%); URF_A1C, 10 (2.2%); URF_UC, 2 (0.4%); URF_A1U, 1 (0.2%); and unclassified, 7 (1.5%). Subtype A1 (n = 141, 62.1%) accounted for the majority of specimens collected in Kenya, followed by subtypes D (n = 34, 15.0%), C (n = 19, 8.4%). Subtypes C (n = 85) and A1 (n = 82) accounted for over 70% of specimens collected in Tanzania, followed by subtype D (n = 42, 17.9%).

### Timeline of the surveys

After the study protocols were approved by institutional Ethical Review Committees and the Office of the Associate Director for Science at U.S. CDC, a total of 1,709 DBS specimens from adult population in the two countries were collected in 3 months and 23 days. A total of 861 DBS specimens from pediatric populations in the two countries were collected in 4 months and 21 days. The DBS specimens from the two countries were shipped to U.S. CDC by World Courier on September 20, 2013 and September 26, 2013, and they were received by the testing laboratory on September 23, 2013 and September 30, 2013, respectively.

Viral load measurement was performed on Kenya specimens on September 25, 2013, by October 8 all VL testing and repeat testing was completed with Kenya specimens by five laboratory personnel. With two NucliSENS® easyMAG® and one EasyQ® instruments, approximately 400 person hours were spent in VL measurement under the condition that all necessary supplies and reagents were in stock. Drug resistance genotyping was initiated one week late, and completed on October 30 by two laboratory personnel using one ABI Prism™ 3730 Genetic Analyzer. Approximately 192 person hours were spent in genotyping of 244 specimens using the ATCC kits.

Viral load measurement on Tanzania specimens was started on October 22. Due to temporary duty travel of two laboratory staffers, it took longer to complete the VL testing for Tanzania specimens, which was done on December 12. Genotyping on Tanzania specimens was then started on December 19 and completed on Jan 23, 2014. It took 1 month and 8 days from specimen receipt to completion of VL testing and DR genotyping for Kenya specimens, and 3 months and 23 days for Tanzania specimens, for which testing was not initiated until about one month after receipt. As a note, 37 DBS specimens from Tanzania with VL ≥1,000 copies/mL failed DR genotyping initially were retested. The repeat DR genotyping was started on May 16, and completed on May 19.

**Fig 4:**
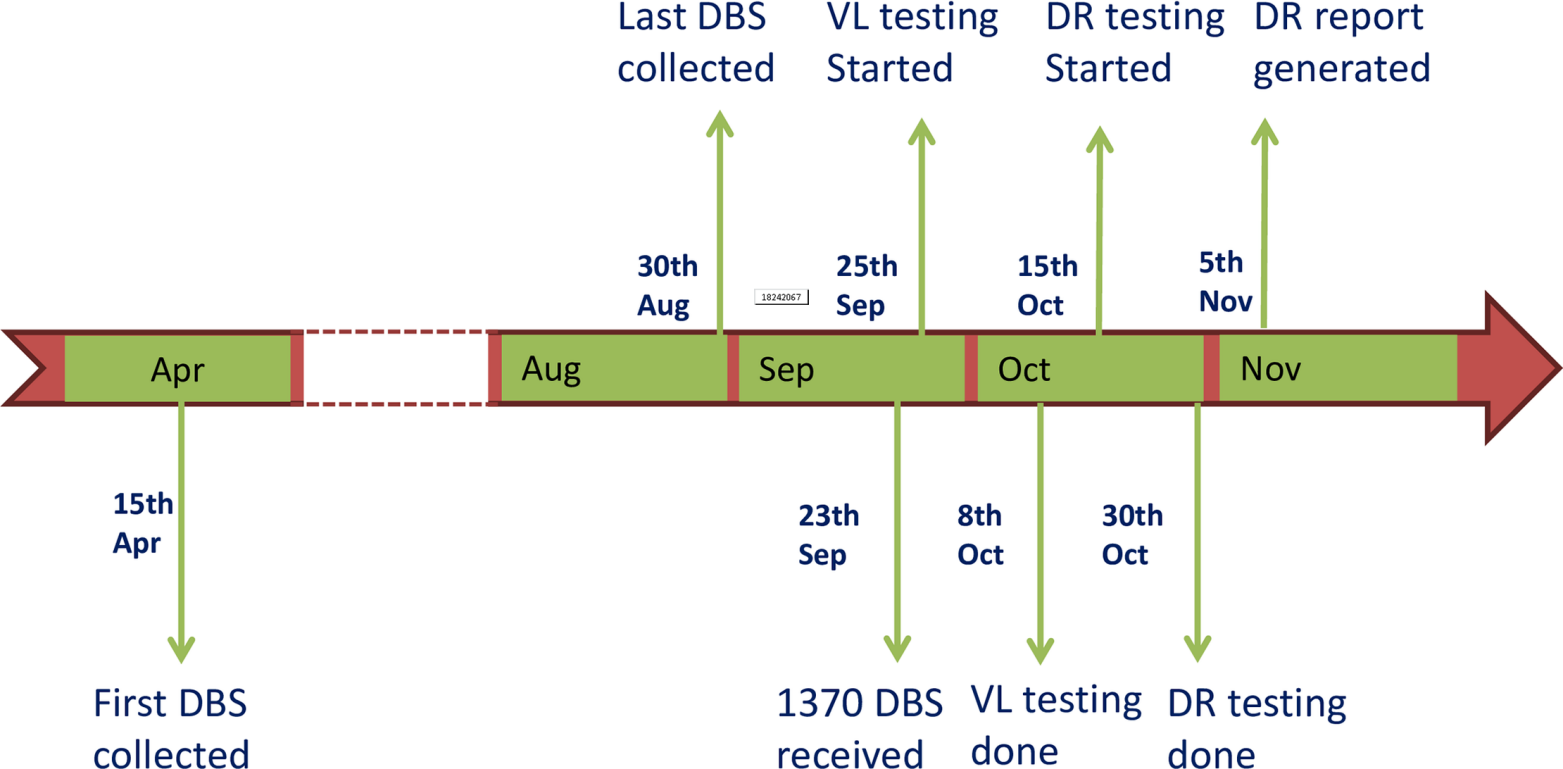
Timeline of nationwide surveillance of acquired HIVDR in Kenya, 2013.

For Kenya, the turnaround time from specimen collection which started on April 15, 2013, until the completion of laboratory testing on October 30, 2013, was 6.5 months. For Tanzania, the turnaround time from specimen collection which started on April 15, 2013, until the completion of laboratory testing on January 23, 2014, was 9.3 months. Kenya survey, in Fig 4, is shown as an example for depicting the timeline of the survey.

## Discussions

To the best of our knowledge, this pilot study represents the first time that DBS sample type-based strategies were applied for nationwide ADR surveys. Our results in Kenya and Tanzania with rapid scale-up of ART have showed that DBS as ADR survey sample type not only made the nationally representative sampling easier and expedited the survey process, it also demonstrated that high genotyping rates could be achieved by more sensitive DR genotyping assay under optimal DBS collection, storage, and shipping conditions, thus meeting the WHO recommended standard for HIVDR surveys in ART populations. The study provides confidence that DBS collected and stored at ambient temperature for two weeks, and shipped to genotyping laboratory with routine courier services are a feasible sample type for large-scale HIVDR surveys.

The high genotyping rates achieved using DBS specimens in this study may be attributed to the following reasons. Firstly, high quality of DBS specimens were prepared by the pre-trained clinical staff, including the optimal DBS collection/storage procedures; secondly, high quality RNA was extracted from magnetic bead-based extraction systems with fewer inhibitors than column-based systems [45, 46]; lastly, broadly sensitive genotyping assay using ATCC HIV-1 Drug Resistance Genotyping kits enhanced the genotyping sensitivity as demonstrated before [40]. In this study, a strong correlation was observed between VL levels and DR Genotyping rates: over 90% of DBS specimens with VL greater than or equal to 5,000 copies/mL, and 80%-85% of DBS specimens with VL between 1,000–4,999 copies/mL were successfully genotyped. Our data demonstrate that a cutoff of 1,000 copies/mL is adequate for HIVDR genotyping on DBS specimens.

DBS are more practical than plasma specimens in sample collection in the countries with limited resources as DBS specimens don’t require cold-chain in sample storage and transportation [26]. According to the WHO guideline, DBS specimens, once completely dried, are considered noninfectious and nonhazardous [47], permitting them to be shipped at ambient temperature using a standard courier service, which significantly reduces the shipment cost. On the other hand, preparing plasma specimens requires laboratory facilities such as centrifuges and cold-chain equipment, including freezers and dry ice. Such facilities are not reliably available in many resource-limited settings, especially in remote or rural areas.

Finally, the cost of the HIVDR survey using DBS can be considerably reduced, as DBS shipment doesn’t require dry ice, and they can be transported at ambient temperature using a standard courier service. In this study the specimen shipment costed approximately $250 per country, whereas the estimated cost of using plasma samples would have been approximately $5,000 per country. In addition, the use of ATCC kit further reduces the cost of HIVDR genotyping, the other commercially available HIVDR genotying kits cost about $200 per test at the time when study was conducted [40], while the reagent cost of the ATCC kit was about $60 per test. With 500 specimens genotyped, $50,000 had been saved in reagent alone.

Because of the convenience of using DBS, the period of collection and shipment of specimens was considerably shortened. The ready to use master mixture in the ATCC kits further expedited the testing process. Furthermore, customized ReCALL software specially modified for ATCC kit genotyping greatly improved the efficiency and quality of sequencing data analysis. The combination of these technologies provides a solution to ensure that nationwide HIVDR surveillance results are available to decision-makers in a timely fashion. We had some of the specimens collected in Tanzania that were exposed to ambient temperatures for more than two weeks, however, no statistically significant decrease in genotyping efficiency was observed. A previous study showed that genotyping efficiency was reduced for the DBS stored at ambient temperature for 4 weeks [26].

The limitations of VL testing and DR genotyping using DBS specimens have been described in detail elsewhere [26, 31, 40, 48]. It has been known that that some sequences obtained from DBS collected from patients with low VL may not fully reflect the circulating viruses, thus can lead to discordant HIVDR interpretations between DBS and plasma specimens. Plasma specimens obtained from whole blood are considered the gold standard specimen type for HIVDR genotyping because HIV sequence data obtained from plasma are derived from actively replicating viruses that are circulating in the body. However many studies have showed a high concordance rate between HIVDR genotypes generated from plasma and DBS in ART-experienced patients [25, 26, 40]. It should be noted that in this study, both VL measurement and DR genotyping were performed in a College of American Pathologists-accredited and WHO-specialized drug resistance laboratory, which is well-equipped and has high standards for QA/QC procedures, reagent and supplies are readily available. However similar studies [32] or HIVDR surveys [9] in countries with limited resources have recently also confirmed the performance and advantages of using DBS sample type in population-based HIVDR surveillance. In addition, only DBS samples were collected for the pilot surveys, which limited our capability to make a comparison between DBS and standard specimen type plasma.

## Conclusions

Simplicity of DBS-based sample collection and reduced cost of in-house or commercially available HIVDR genotyping assays make timely and large-scale/nationwide surveillance of ADR feasible. Results from our pilot study indicate that cross-sectional surveillance of ADR using DBS in combination with ATCC kits, currently manufactured by ThermoFisher Scientific (The HIV-1 Genotyping kits, https://www.thermofisher.com/us/en/home/life-science/sequencing/sanger-sequencing/applications/genotyping-hiv-detect-drug-resistance.html) and ReCALL software is cost-effective and time-saving methodology. It is practical to reduce the field sampling to a maximum of six months in a nationwide survey, as recommended by the WHO protocol for surveillance of HIV drug resistance in patients receiving ART [49]. Laboratory testing turnaround time can be as short as two months when a validated DBS genotyping assay is applied.
